# Plastic Waste Management Strategies and Their Environmental Aspects: A Scientometric Analysis and Comprehensive Review

**DOI:** 10.3390/ijerph19084556

**Published:** 2022-04-10

**Authors:** Saimin Huang, Hongchang Wang, Waqas Ahmad, Ayaz Ahmad, Nikolai Ivanovich Vatin, Abdeliazim Mustafa Mohamed, Ahmed Farouk Deifalla, Imran Mehmood

**Affiliations:** 1 College of Civil Engineering, Nanjing Forestry University, Nanjing 210037, China; njly18068676995@sina.com; 2 Department of Civil Engineering, COMSATS University Islamabad, Abbottabad 22060, Pakistan; ayazahmad@cuiatd.edu.pk; 3 Peter the Great St. Petersburg Polytechnic University, 195291 St. Petersburg, Russia; vatin@mail.ru; 4 Department of Civil Engineering, College of Engineering in Al-Kharj, Prince Sattam bin Abdulaziz University, Al-Kharj 11942, Saudi Arabia; a.bilal@psau.edu.sa; 5 Building & Construction Technology Department, Bayan College of Science and Technology, Khartoum 11115, Sudan; 6 Structural Engineering Department, Structural Engineering and Construction Management, Future University in Egypt, Cairo 11835, Cairo Governorate, Egypt; ahmed.deifalla@fue.edu.eg; 7 Department of Building and Real Estate, The Hong Kong Polytechnic University, Hung Hom, Kowloon, Hong Kong 999077, China; imran.mehmood@connect.polyu.hk

**Keywords:** environmental pollution, plastic waste, plastic waste recycling, waste management, scientometric analysis

## Abstract

Plastic consumption increases with the growing population worldwide and results in increased quantities of plastic waste. There are various plastic waste management strategies; however, the present management progress is not sustainable, and plastic waste dumping in landfills is still the most commonly employed strategy. Being nonbiodegradable, plastic waste dumping in landfills creates several environmental and human health problems. Numerous research studies have been conducted recently to determine safe and ecologically beneficial methods of plastic waste handling. This article performed a bibliographic analysis of the available literature on plastic waste management using a computational approach. The highly used keywords, most frequently cited papers and authors, actively participating countries, and sources of publications were analyzed during the bibliographic analysis. In addition, the various plastic waste management strategies and their environmental benefits have been discussed. It has been concluded that among the six plastic waste management techniques (landfills, recycling, pyrolysis, liquefaction, road construction and tar, and concrete production), road construction and tar and concrete production are the two most effective strategies. This is due to significant benefits, such as ease of localization, decreased greenhouse gas emissions, and increased durability and sustainability of manufactured materials, structures, and roadways. Conversely, using landfills is the most undesirable strategy because of the associated environmental and human health concerns. Recycling has equal benefits and drawbacks. In comparison, pyrolysis and liquefaction are favorable due to the production of char and fuel, but high energy requirements limit their benefits. Hence, the use of plastic waste for construction applications is recommended.

## 1. Introduction

Industrial growth results in a huge number of goods for human activities and a massive quantity of waste in the environment as a result of used items being discarded following human activities. These wastes include gaseous, liquid, and solid wastes [1,2,3]. Plastic waste (PW) is a typical industrial waste, and its disposal into landfills creates serious environmental concerns [4,5,6]. Plastic items have become an essential part of people’s everyday lives and are utilized in a variety of sectors, including construction, healthcare, electronics, agriculture, the automotive industry, and packaging [7]. Plastic demand continues to grow due to its numerous advantages, including resistance to erosion, durability, convenience, simplicity of production, and cheap cost [8,9]. Worldwide plastic output has increased at an annually compounded rate of 8.4% since 1950; plastic manufacture reached 0.36 billion tons in 2018 and is expected to surpass 0.50 billion tons in 2025 [10,11,12]. Around 60% of PW is not recycled and encounters the environment [13]. The primary plastics in demand include polyvinyl chloride (PVC), polyethylene (PE), polyethylene terephthalate (PET), polypropylene (PP), polyurethane (PUR), and polystyrene (PS) [14]. Similarly, PW is derived from the aforementioned key plastic types. For instance, the packaging sector consumes the most plastics produced and is the primary source of PW due to the extensive usage of disposable items [12]. PW discarded by the packaging industry is mostly composed of PET, PS, PE, and PP [15]. PW degrades slowly and can persist in the environment for hundreds of years; it is thus referred to as nonbiodegradable waste. These industries contribute to the ever-growing quantity of global nonbiodegradable PW, resulting in a variety of environmental challenges [16].

**Figure 1:**
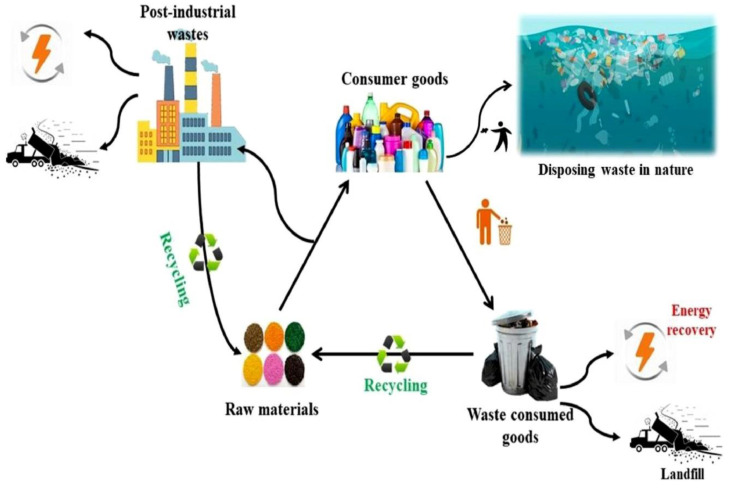
Plastic products’ life cycle [32].

Recycling is a promising option for lowering the demand for new raw materials and reducing waste in landfills [17,18,19]. Additionally, waste recycling benefits the environment by minimizing pollution caused by raw material extraction, conserving energy, and even providing domestic jobs [20]. Figure 1 shows the life cycle of plastic products from consumption to recycling/disposal. Numerous approaches have been developed and utilized in the management of nonbiodegradable PW to date. These technologies may be classified into two categories: traditional PW and advanced PW treatment. The conventional methods of PW disposal are incineration and landfilling, both of which are constrained by a specific bottleneck. Incinerating PW consumes substantial energy and produces harmful by-products [21]. CO_2_, persistent organic compounds, particulate matter, acidic gases, and heavy metals are all extremely hazardous by-products that contribute to global warming and a variety of health issues, including lung function problems, respiratory symptoms, and an increased risk of cancer [22,23]. Landfilling has historically been used to handle PW, and even today, the majority of PW is disposed of in landfills [24,25]. Nonbiodegradable PW, on the other hand, decomposes slowly under landfill settings, requiring a growing quantity of land due to the rising volume of PW disposal. Additionally, the contact of PW with groundwater and harmful compounds soluble in water in landfills can result in the production of toxic leachate, which can cause the deterioration of the surrounding soil [26]. As a result, landfilling has been deemed an extremely adverse managing option and has been subject to a variety of restrictions [27]. To address the limitations of conventional methods, such as incineration and landfilling, a variety of innovative plastic management strategies have been established, including pyrolysis, liquefaction, and construction applications [28,29,30,31].

As research on PW management expands due to the growing trend towards environmental protection, scholars confront information burdens that could hinder creative exploration and academic partnerships. It is therefore necessary to devise and implement a technique that enables scholars to obtain crucial information from the most dependable sources possible. A scientometric approach may aid in alleviating this weakness via a software application. The goal of this study is to undertake a scientometric analysis of bibliographic data published up to 2021 on PW management and a review of the different management strategies for PW that help reduce environmental pollution. A scientometric analysis can perform the quantitative evaluation of vast bibliometric data using a suitable software application. Conventional review studies are deficient in providing a complete and correct connection between disparate portions of the literature. Science mapping, co-occurrence, and co-citation are among the extremely difficult aspects of current exploration. The scientometric study includes the detection of the sources with the most articles, keyword concurrence, the leading authors in terms of papers and citations, and the regions actively participating in researching PW management. The Scopus database was used to extract bibliometric data for 6,101 relevant papers, which were then analyzed using VOSviewer software. Furthermore, the impact of various management strategies for PW on the environment was discussed. The aim was to analyze the benefits and drawbacks of each management technique from the literature and suggest the most desirable ones.

## 2. Scientometric Analysis Methods

This study employed scientometric analysis [33,34,35,36,37] along with a traditional review for the quantitative evaluation of the different aspects of the bibliographic data. Numerous articles have been published on the subject matter, and it is very important to pick a highly reliable search engine. Scopus and the Web of Science are two highly accurate search engines for that purpose [38,39]. The bibliographic data for the present study on PW management was collected from Scopus, as it is commonly recommended by scholars [38,40,41]. The search term “plastic waste management” returned 7756 articles in the Scopus database as of March 2022. To exclude unnecessary papers, various filter preferences were employed. The classifications “article”, “review”, “conference paper”, and “conference review” were chosen as the “document types”. The “source kind” was selected as “journal” and “conference proceeding”. The “publishing year” constraint was chosen to include papers published up through “2021”, and the selected “language” was “English”. Subsequent to the application of these conditions, 6,101 records were retained. The same procedure was also reported by various scholars [42,43,44,45].

**Figure 2:**
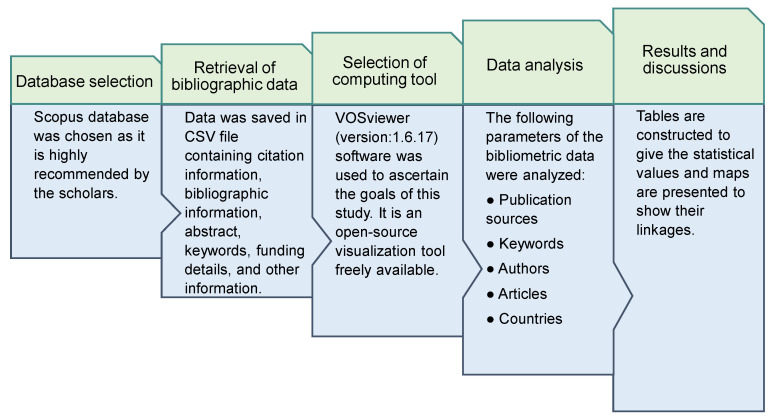
Flowchart of the analysis procedure.

Scientometric studies use science mapping, a method invented by researchers for interpreting bibliometric records for a number of reasons [46]. Data from Scopus was stored in the comma separated values (CSV) format for further assessment by employing compatible software. For further analysis, VOSviewer (version 1.6.17, Leiden University, Leiden, The Netherlands) was utilized to construct the scientific visualization and quantitative assessment of the literature. VOSviewer is an open-source and freely available visualization product that is broadly employed in a range of disciplines and well-reviewed by scholars [47,48,49,50,51]. Thus, the current study’s objectives were met by utilizing VOSviewer. In VOSviewer, the retrieved CSV files were imported, and further analysis was carried out while keeping data consistency and reliability. The sources of publications, the most frequently used keywords, the authors with the most citations, and countries’ participation were evaluated during the bibliographic evaluation. The several aspects, their associations, and co-occurrence were visualized through diagrams, while their statistical values were listed in tables. Figure 2 demonstrates the flowchart of the scientometric review.

## 3. Results and Discussions

### 3.1. Subject Areas and Annual Publication Pattern

**Figure 3:**
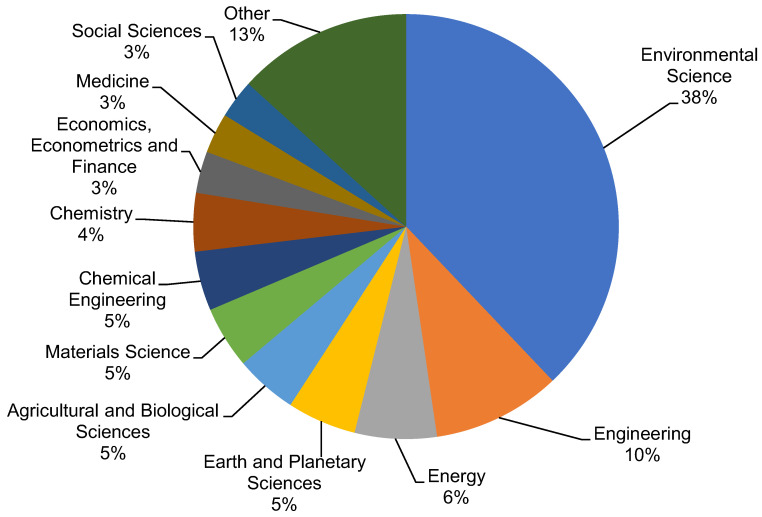
The subject areas of the articles.

**Figure 4:**
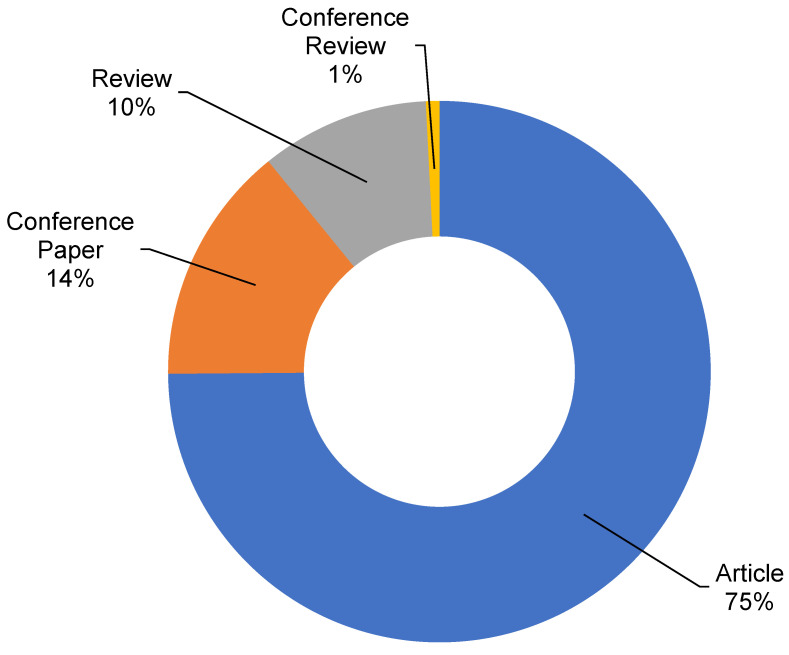
Various types of documents published in related fields of study.

**Figure 5:**
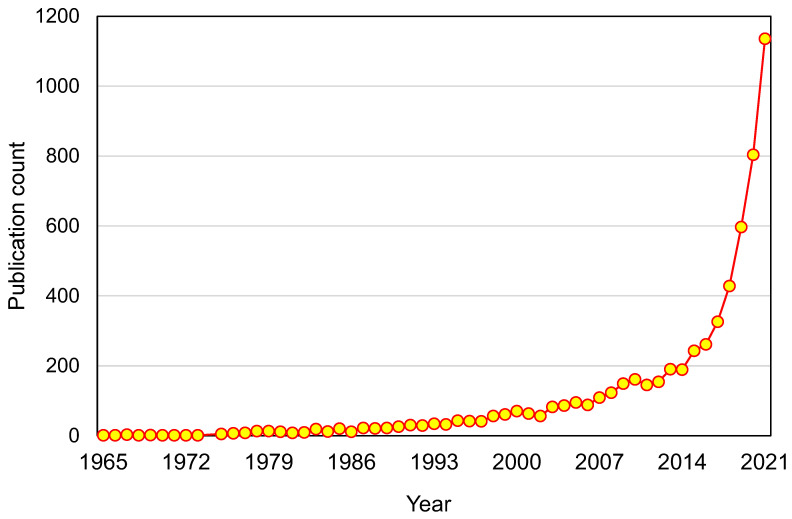
Annual publication trend for articles.

The Scopus analyzer was employed to perform such analysis as to determine the most relevant research areas. The three leading document-producing areas were found to be Environmental Science, Engineering, and Materials Science, as displayed in Figure 3. For the searched terms “plastic waste management”, publications of the Environmental Science, Engineering, and Materials Science disciplines comprised around 38%, 10%, and 6% of the documents, respectively, accounting for a total of 54% contribution based on the document count. Furthermore, the type of documents was also analyzed for the searched keywords in the Scopus database, as depicted in Figure 4. This analysis revealed that, for “plastic waste management”, journal articles, conference papers, journal reviews, and conference reviews comprised nearly 75%, 14%, 10%, and 1% of the total documents, respectively. Figure 5 illustrates the annual trend of publications in the present study area from 1965 to 2021, as the first document on the subject research area was found to have been published in 1965. Steady growth in the publication count was noticed, with an average of around 19 articles annually up to 2000. This annual average of publications rose to about 129 from 2001 to 2015. The number of publications rose substantially in the last six years (2016–2021), with an average of around 592 publications per year.

### 3.2. Publication Sources

**Figure 6:**
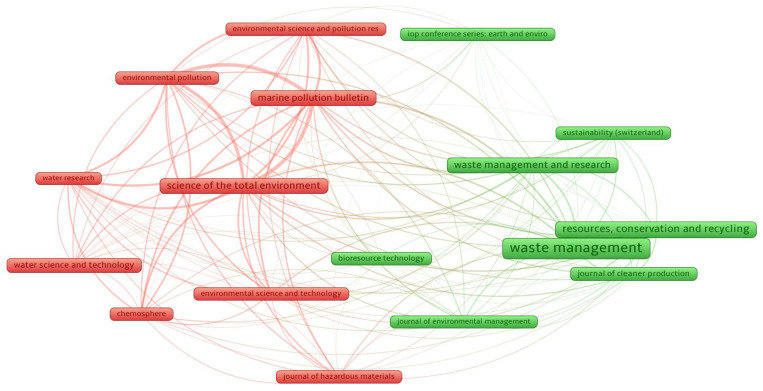
Science mapping of publication sources with a minimum of 50 articles in the relevant study area. Box size is proportional to the number of articles published in a particular journal, and color refers to the clustering of journals based on co-citation patterns in the published articles.

This investigation was carried out on the retrieved bibliometric data with the VOSviewer. The “analysis type” was selected as “bibliographic coupling”, and the “analysis unit” was kept as “sources”. A threshold of at least 50 papers per source was established, and 17 of the 1555 sources satisfied these constraints. The publication sources (journals) that published a minimum of 71 papers, including data on PW recycling from up to 2021, are displayed in Table 1, together with their number of citations received in that time period. *Waste Management; Resources, Conservation and Recycling,* and *Science of the Total Environment* are the leading three sources/journals in terms of the number of papers, with 548, 270, and 227 papers published, respectively. Moreover, the aforementioned journals also received the most citations in the related research area with 25,171, 13,142, and 11,555 citations, respectively. Notably, this analysis would provide a basis for forthcoming scientometric studies in this subject area. In addition, past manual evaluations were unable to provide science mapping visualization maps. Figure 6 represents a visualization map of the journals that have published at least 71 documents. The box size is proportional to the journal’s article count in the current research; a bigger box size implies a larger role. As an example, *Waste Management* has larger box dimensions than the others, denoting that it is a highly significant journal in that area. Two clusters were generated, each represented by a different color (red and green) in the illustration. Clusters are constructed depending on the extent of research sources or the frequency with which they are cited together [52]. In this analysis, clusters are formed based on the frequency with which they are cited together. The VOSviewer constructed clusters of journals identified by varying colors based on co-citation patterns in the published articles. For example, the green cluster comprises eight journals that are repeatedly cited in similar articles. Moreover, closely-spaced frames (journals) have stronger connections than widely spread frames. As an example, *Waste Management* is more directly related to *Resources, Conservation and Recycling* than it is to the *Journal of Environmental Management.*

### 3.3. Keywords

**Figure 7:**
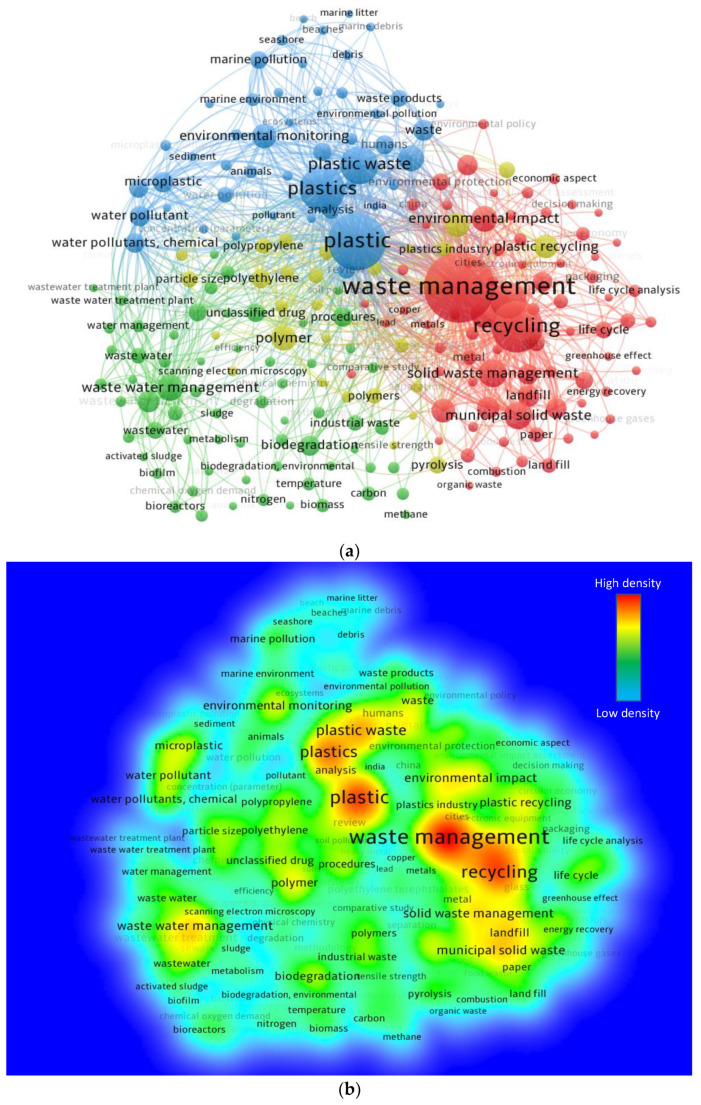
Co-occurrence of keywords: (**a**) Scientific mapping: circle size is proportional to the number of co-occurrences of a particular keyword; different colors show distinct clusters (red: cluster 1, green: cluster 2, blue: cluster 3, and yellow: cluster 4), and clustering by color is based on patterns of co-occurrence among multiple keywords in the published articles; (**b**) Density mapping.

Keywords are crucial in research as they define and indicate the basic topic of the study domain [53]. For the analysis, the “analytical type” was selected as “co-occurrence” and the “analysis unit” as “all keywords”. The lowest number of occurrences for a keyword was kept 100. As a result of these limits, 221 of the 36,091 keywords satisfied the condition. The leading 20 keywords that were employed frequently in the published papers in the subject area are displayed in Table 2. The top five most frequently occurring keywords on the subject topic include “waste management”, “plastic”, “recycling”, “plastics”, and “plastic waste”. “Plastics” and “plastic” are the same keywords and need to be considered as a single keyword. However, no such option in the VOSviewer is available to merge the “plastic” and “plastics” keywords. So, they were treated separately. We might add their numerical values manually (2290 + 1548 = 3838), but they will not be displayed in the figure. This analysis revealed that PW recycling has been under study most often for waste management and sustainable development. Figure 7 shows the visualization map of keywords in terms of their co-occurrences, their links, and the density connected with their rate of recurrence. The dimension of a keyword circle in Figure 7a reflects its frequency, while its position implies its co-occurrence in articles. Furthermore, the image demonstrates that the specified keywords have greater circles than the others, signifying that they are vital keywords in the research of PW management. Clusters of keywords have been highlighted distinctively in the graph to reflect their co-occurrence across a variety of publications. Clustering by color is based on patterns of co-occurrence among multiple keywords in the published articles. Green, red, blue, and yellow suggest the presence of four clusters (Figure 7a). As seen in Figure 7b, diverse colors correspond to different levels of keyword density. The colors are ordered by their density, with red having the maximum and blue having the minimum density. “Waste management”, “plastic”, “recycling”, and “plastics” have red areas, suggesting a greater density. This finding will assist future writers in picking keywords that will expedite the discovery of published data in a specific area.

### 3.4. Authors

**Figure 8:**
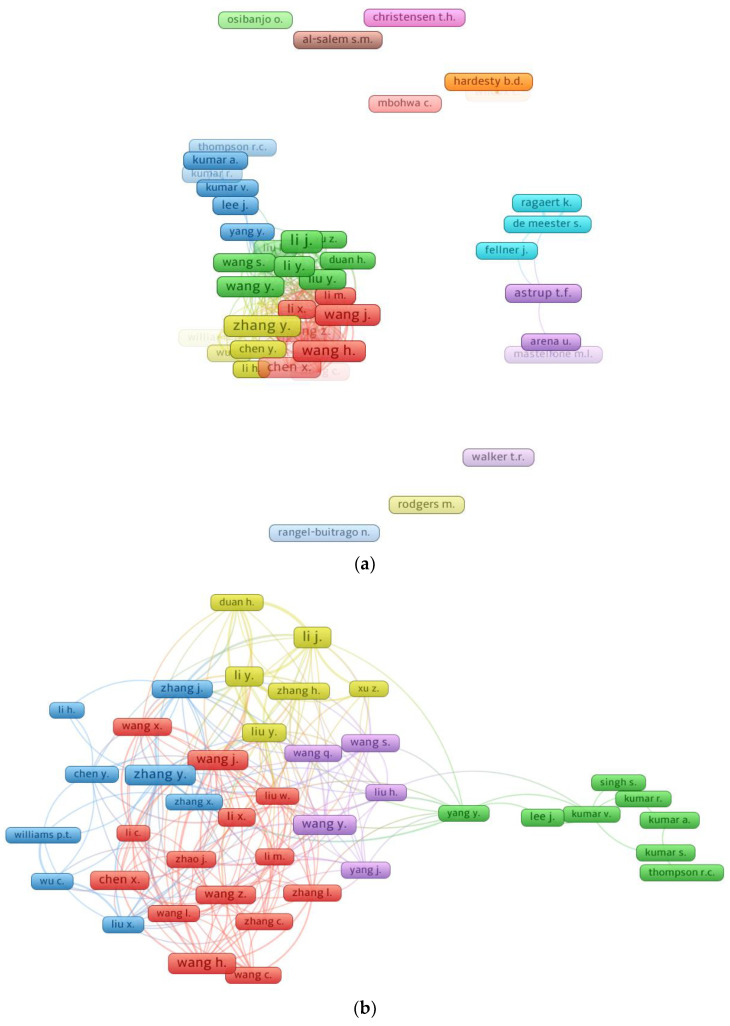
Science mapping of authors: (**a**) With a minimum of 10 publications; (**b**) Linked authors based on citations. Box size is proportional to the number of publications by a particular author, and color refers to the clustering of authors based on patterns of co-citation in the publications.

Citation numbers show how influential a researcher is in a given research domain [54]. In the VOSviewer, “co-authorship” was chosen as the “analysis kind”, and “authors” as the “analysis unit”. The fewest number of papers required for a writer was set at 10, and 60 of the 18,236 writers satisfied this criterion. Table 3 displays the leading writers in terms of publications and total citations in the research of PW management, as assessed by data obtained from the Scopus search engine. The average number of citations for each author was computed by dividing the total number of citations by the total number of papers. It was complicated to measure a scientist’s efficacy considering all aspects, such as the number of publications, total citations, and average number of citations. Conversely, the writer’s evaluation was defined by equating each aspect independently, i.e., total publications, total citations, and average citations. When it comes to overall papers, the leading three authors are Li, J. with 39; Wang, H. with 36; and Zhang Y. with 34 papers. When it comes to total citations, Thompson, R.C. takes the lead with 7055; Wilcox, C. is second with 5130; and Li, J. is third with 1964 total citations in the present study area. Furthermore, when comparing average numbers of citations, the following authors come out on top: Thompson, R.C. has about 588; Wilcox, C. has about 395; and Al-Salem, S.M. has about 140 average citations. The visualization of writers with at least 10 papers and the connection of the most notable authors is shown in Figure 8. It was noticed that 41 of the 60 authors were connected based on citations. This analysis disclosed that several authors are interconnected based on citations in the research of PW management.

### 3.5. Documents

**Figure 9:**
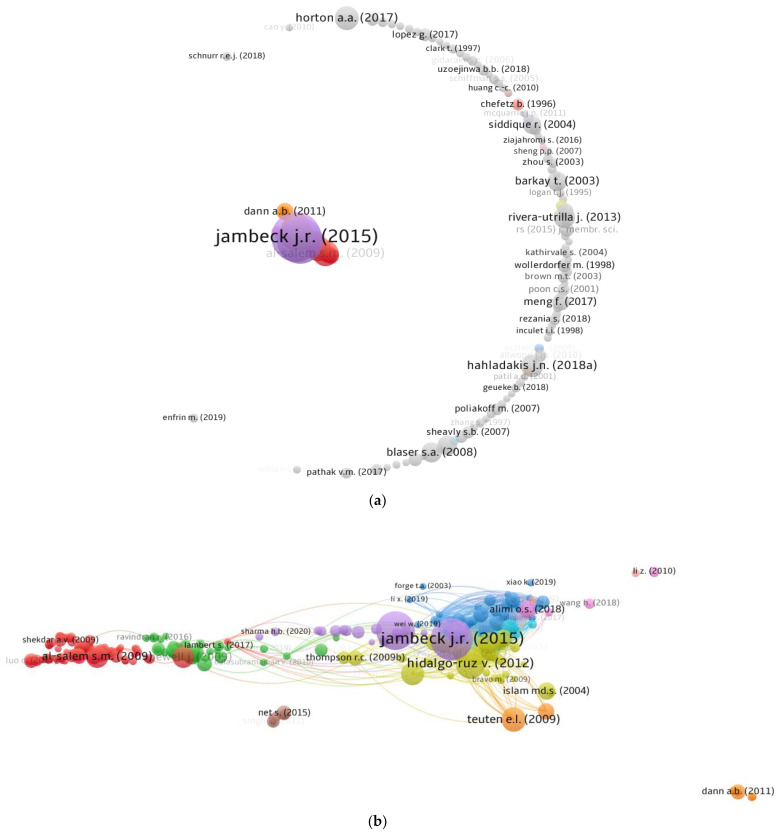

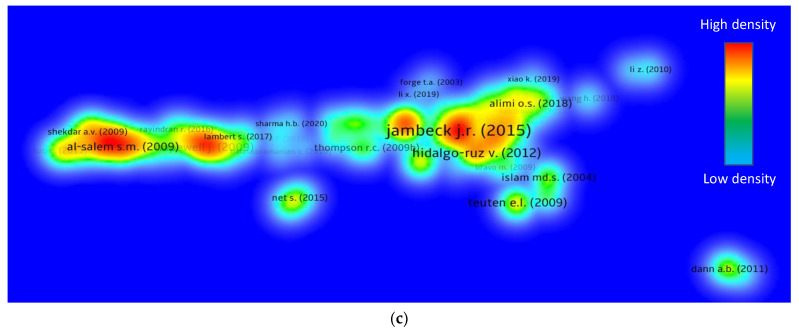
Scientific visualization of documents: (**a**) Documents with a minimum of 100 citations; (**b**) Linked documents based on citations: circle size is proportional to the number of co-occurrences of a particular keyword, and different colors show distinct clusters formed based on the pattern of co-citations; (**c**) Density of linked documents.

The number of citations a document receives indicates its influence within a specific field of research. Citation-dense papers are often regarded as pioneers in their respective fields of research. For this assessment, “analysis kind” was set as “bibliographic coupling” and “analysis unit” as “documents”. The limit for the lowest number of citations set to 100, and 384 of the 6101 documents met this criterion. The leading 10 articles based on citations in the field of PW management are given in Table 4, along with their writers and citations details. Jambeck, J.R. [55] obtained 4313 citations for their publication “Plastic waste inputs from land into the ocean”. Geyer, R. [13] and Hidalgo-Ruz, V. [56] received 3675 and 2007 citations, respectively, for their particular works and were rated in the leading 3. However, only 10 articles received more than 1000 citations up through 2021. Additionally, Figure 9 demonstrates the writer’s mapping of documents, connections based on citations, and the density of the connected documents in the current study topic. The VOSviewer study determined that 286 of 384 documents were connected based on citations. Figure 9a illustrates the scientific visualization of the contributors to the study of the topic under study whereas Figure 9b represents the mapping of connected documents based on citations. Furthermore, the density mapping (Figure 9c) demonstrates the increased density concentration of the top articles.

### 3.6. Countries

**Figure 10:**
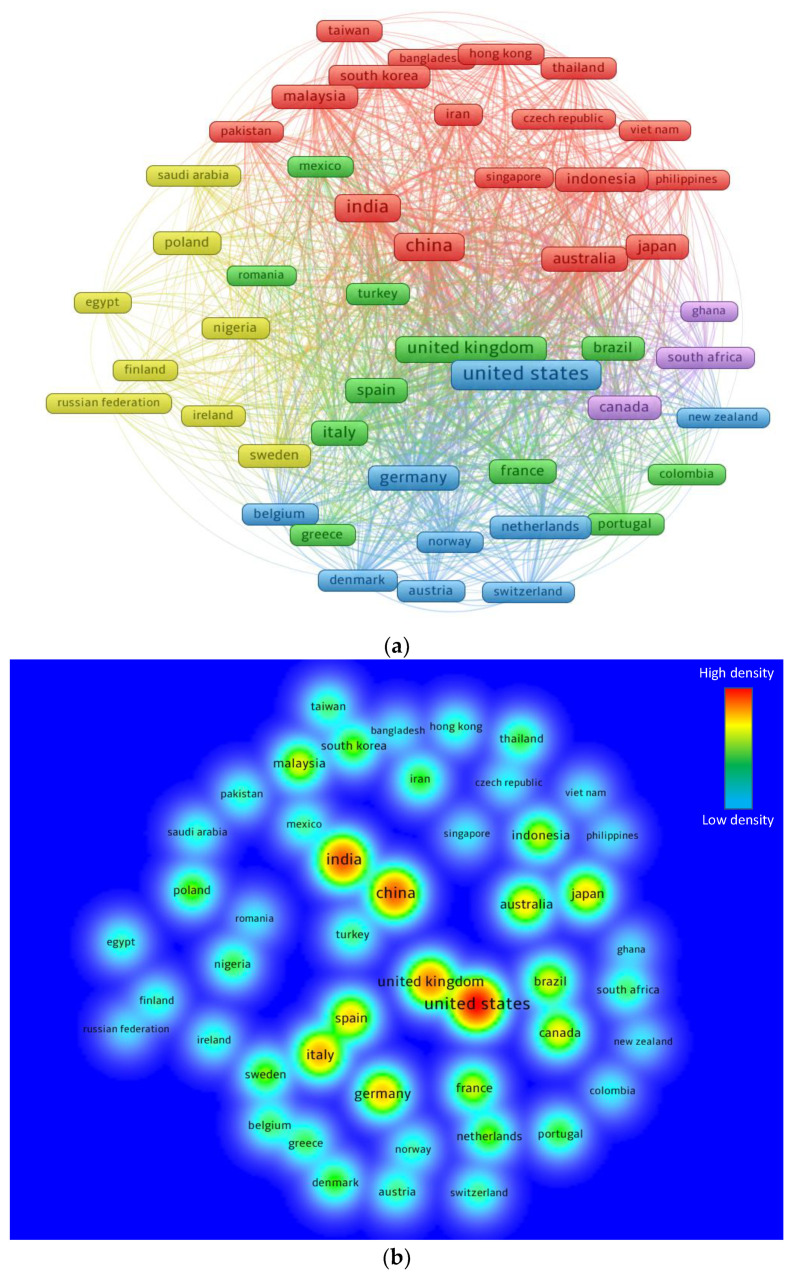
Science mapping of the top contributing countries: (**a**) Network visualization: box size is proportional to the number of publications by a particular country, and different colors show distinct clusters created based on the pattern of co-citations of countries in published articles; (**b**) Density visualization.

Different nations have provided more to the current research area than others have and intend to. The network mapping was established to enable readers to view locations dedicated to eco-friendly construction. The “analysis type” was selected as “bibliographic coupling”, and the “analysis unit” as “countries”. The lowest document limit for a nation was set 30, and 48 countries satisfied this condition. Table 5 shows the top 20 countries based on publications in the current research area. The United States, India, and China presented the most papers with 871, 581, and 551 documents, respectively. However, the United States has the highest total number of citations at 42,924, then the United Kingdom with 30,071, and China with 19,944 total citations. Figure 10 depicts the science mapping visualization as well as the density of nations connected by citations. The size of a box is comparative to the impact of a nation on the subject topic (Figure 10a). According to the density visualization, the nations with the highest involvement had a greater density (Figure 10b).

## 4. Management Techniques for Plastic Waste

**Figure 11:**
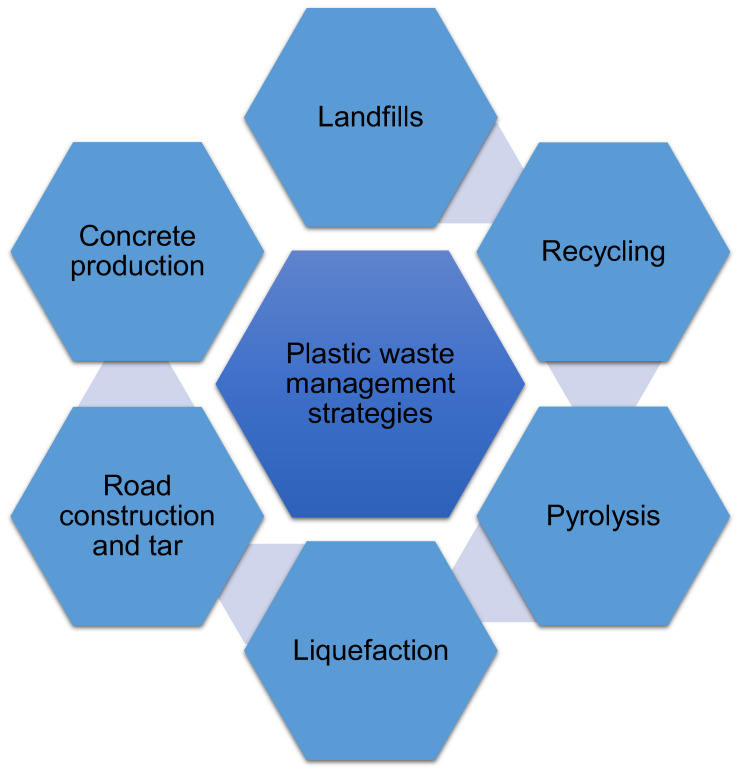
Various management techniques for plastic waste.

The management techniques for PW are broadly classified into six categories, namely landfills, recycling, pyrolysis, liquefaction, road construction and tar, and concrete production [63], as displayed in Figure 11. These techniques are briefly discussed in the subsequent subsections.

### 4.1. Landfills

**Figure 12:**
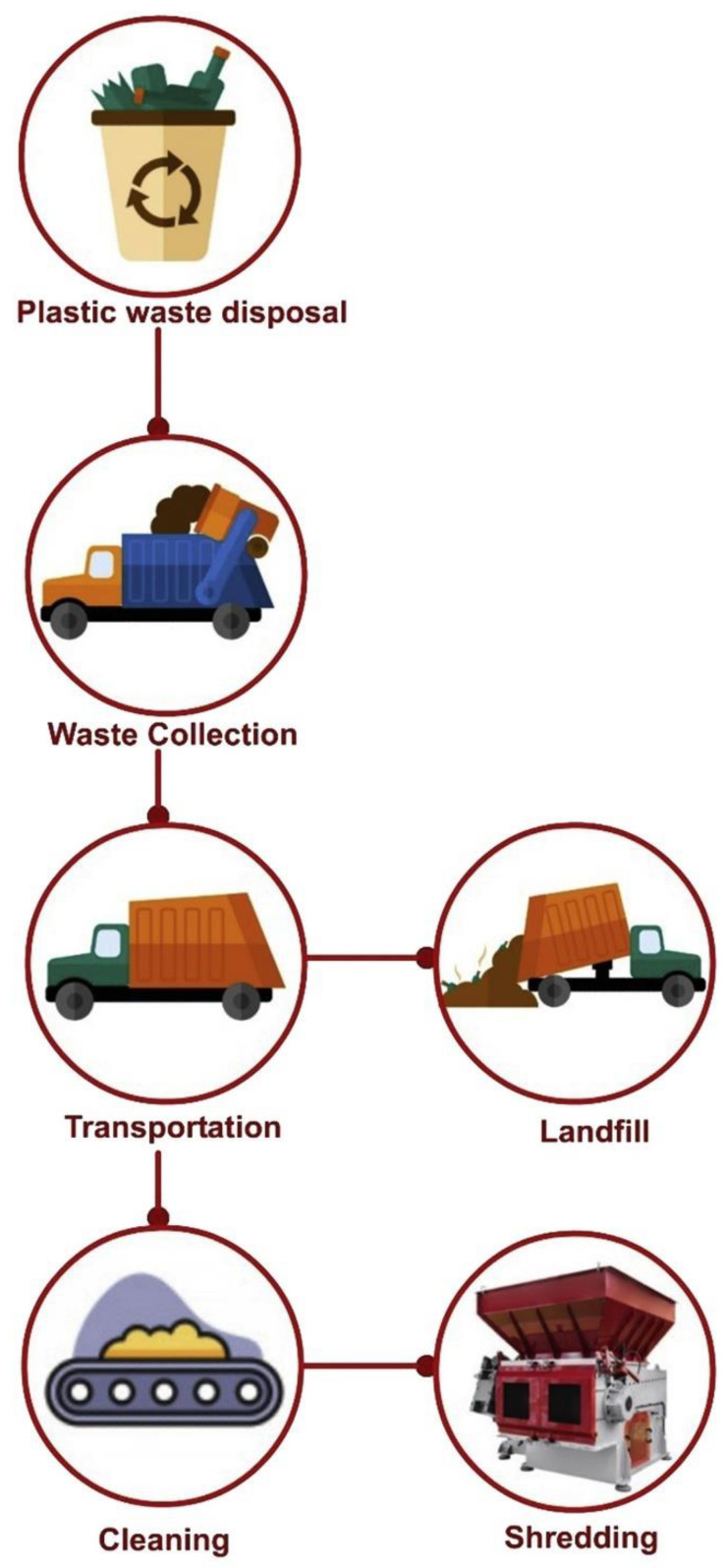
Flowchart of plastic waste management from generation to landfill/shredding [63].

**Figure 13:**
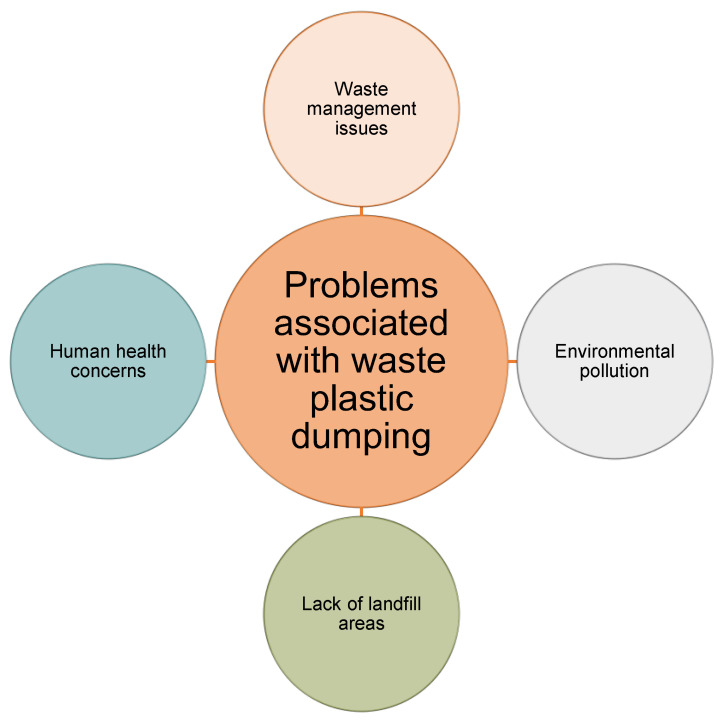
Disadvantages of plastic waste disposal in landfills.

Landfilling is the most rudimentary technique of PW disposal. Landfills contain a great deal of garbage and have been linked to a number of issues. It is not a sustainable means of disposing of PW [64]. The process of landfilling PW from generation to disposal is shown in Figure 12, and Figure 13 illustrates the issues connected with the disposal of PW based on a review of the literature. Disposing of PW in landfills may exacerbate land shortages and hinder the operations of waste management organizations [5,65,66]. Additionally, when PW encounters bodies of water, it contaminates them [67,68]. Hence, dumping PW creates concerns for human health and the environment [69]. Landfills have long been recognized as contaminating the soil [68,70]. Thus, landfilling PW must be avoided, and other management techniques should be followed as described in the following sections to help protect the environment.

### 4.2. Recycling

Generally, recycling is the procedure through which PW is re-extruded. PW is mostly recycled mechanically, which is one of the most cost-effective methods [71,72]. The first phase is shredding or cutting, which involves cutting PW using saws or shears into tiny fragments that are simpler to carry. In the contaminant-separation process, paper pieces, dirt, and smaller particles are removed from PW with the help of a cyclone separator. PW with varying densities is separated using a flotation method in order to manage plastic with varying densities. The next step is milling, which collects and mills the individual polymers. Without the pre-processing phases stated prior to milling, the plant’s efficiency is reduced. Following that, the milled PW is cleaned with water. Chemical washing is also useful for some types of material handling (most notably for removing adhesive from PW), where caustic soda and wetting agents are utilized. The materials are then collected and kept or transferred for further processing during the agglutination process. Extrusion of the plastic results in the formation of strands, which are subsequently pelletized to create a single-polymer plastic. The items are quenched by cooling them with room temperature water. Granulated plastic is then offered on the market as grocery bags, blinds, shutters, and other home items [63].

Recycling is based on the notion of remolding plastic material. It is difficult to turn the full amount of PW into a reusable product. This mass reduction throughout the recycling process is accounted for as plastic emission [73]. Another downside of recycling is the significant amount of energy consumed throughout the process [74]. In comparison to the initial product, the durability of these items is significantly reduced. However, the most prudent course of action regarding plastic is to drastically minimize its usage and reliance. Guidelines limiting the usage of recycled materials have been enacted. For example, used PET containers cannot be utilized to package drinks [75]. The manufacture of wood–plastic composites is one recycling process that has gained increasing interest [76]. This approach of PW management entails the creation of novel materials by mixing PW with woody waste biomass in various amounts. The primary advantages of this technology are the capacity to regulate the characteristics of the materials generated and the efficiency with which two distinct forms of waste may be removed in the same process.

Panels made using a mixture of macadamia shells and automotive PW have exhibited more strength and resistance to fire than panels made entirely of automotive PW [77]. Similarly, it was discovered that when PW was combined with straw flour, its mechanical characteristics greatly improved [78]. It was also reported that the field of PW and woody biomass composites is still in its initial stages and needs additional investigations before its long-term viability can be determined.

### 4.3. Pyrolysis

The pyrolysis of PW has been investigated as a method for converting home and industrial PW to fuel by subjecting it to severe process conditions, most notably elevated temperature [79]. It entails the degradation of polymeric plastic molecules with a high molecular weight into light gas and liquid hydrocarbons in the absence of oxygen to avoid the creation of oxygen-containing by-products, such as sulfur and carbon oxides, in a reactor devised to endure severe conditions [79]. There are two primary ways by which it is carried out, which are characterized by the presence or absence of a catalyst. Thermal pyrolysis comprises the application of high temperatures and pressure to PW, resulting in the destruction of the molecule by a mixture of scission, in which the carbon chain is cracked around the center, chain crosslinking, and cyclization of linear structures [80]. On the other hand, catalytic pyrolysis utilizes a catalyst to boost the efficiency of the degradation and decrease the energy needs. The outputs of PW pyrolysis vary according to the kind and amount of feedstock utilized and the reactor employed. These may be classified into three distinct classes, including gaseous and liquid hydrocarbons and char. The pyrolysis liquid products are essential and can be utilized as a direct fuel or after mixing with gasoline, motor oil, or diesel, provided they sustain necessary characteristics [81].

The most-often obtained liquid products are paraffin (octane, heptane, and butane), olefins, isoparaffins, propane, and aromatics [82]. Char is a carbonaceous, solid substance that is produced as a by-product of the manufacturing of liquid oil and natural gas. Increases in the proportion of char generated were found to be associated with increases in the pyrolysis temperature [83]. Pyrolysis produces gaseous products mostly composed of lighter hydrocarbons formed by successive successful cracking, as well as some volatile impurities from char. The yield of gaseous products from the pyrolysis of PW, including low-density polyethylene (LDPE) and polystyrene and is much less than that of liquid products, with an increase in gaseous yield being proportional to a rise in temperature [84]. In comparison, polyvinyl chloride and polyethylene terephthalate-based PW yielded substantial gases (>75% by weight) because of their different processes and low energy requirements [85] These gaseous products (methane, butane, ethane, and propane) can be used to generate electricity.

Temperature has the greatest effect on a pyrolysis reaction of PW, with the endothermic characteristic of pyrolysis resulting in enhanced rates of conversion [86], as well as considerations for extreme operating settings [87]. Additionally, high temperatures were shown to be favorable for cyclization reactions between the products [88] but detrimental to the yield of waxes [89]. Residence time also influences the products, with greater residence time being associated with an increase in the fraction of linear hydrocarbon products and the conversion rate [8]. Pyrolysis of PW can also be carried out in conjunction with biomass, with a range of synergistic effects on the type and yield of the products, including a decrease in the formation of tar and an increase in the liquid yield [82,90]. Microwave pyrolysis is another method that has gained popularity. It entails causing the breakdown of polymeric molecules using high-intensity microwaves that raise the molecules’ surface temperature [91]. The plastic material is turned into a fuel made up of a mixture of hydrocarbons during the pyrolysis of PW.

### 4.4. Liquefaction

Hydrothermal liquefaction has a prolonged history of being used to convert biomass, primarily of algal origin, to bio-oil. It entails the transformation of cellular material into valuable liquid fuel. The method has been adapted to absorb PW and is particularly appealing since it allows for the recovery of plastic for reuse alongside liquid gasoline [92]. Typically, PW is liquefied in the presence of a biomass source, a process known as co-liquefaction. In comparison to alternative waste to value technologies, liquefaction of biomass results in a more even distribution of components among the products. Higher carbon content in the products should naturally result in improved fuel performance [93]. It is worth noting that polymers may be liquefied in the absence of biomass although this is a less popular process than co-liquefaction. For any liquefaction method, catalyst choice is critical and has a significant impact on the process’s efficiency. In most situations, heterogeneous catalysts are used in the liquefaction process to reduce the likelihood of corrosion and to optimize the reaction’s interactions [94].

Depolymerization, disassociation, and recombination are the three processes in the operation of a hydrothermal liquefaction system. Depolymerization of the three major components found in biomass into smaller molecules such as proteins, tiny carbohydrates, and amino acids occurs when a solvent and/or catalyst are added, as well as in high temperature and high pressure conditions [95]. The smaller molecules dissociate further to produce components in their native state, which then recombine to form the product. Bio-oil is created when recombination is combined with a polymerization event. The creation of char and coke arises from further polymerization and degradation of the bio-oil. The lack of a polymerization step leads to the production of biogas [96]. The use of polymers in liquefaction tends to reduce the amount of coke produced. As a result, using plastics in liquefaction can be thought of as having two benefits: recycling PW and improving existing liquefaction products [97]. It has been shown that liquefaction requires a huge amount of water, which might be considered a drawback. Liquefaction is likewise a high-temperature process that consumes a lot of energy [98].

### 4.5. Road Construction and Tar

Tar is an organic compound with a variety of different structures and compositions. Tar is produced in significant amounts when PW is co-gasified or co-pyrolyzed with other compounds such as heavy metals [99,100]. The extremely condensable behavior of the organic components of tar makes its existence in gas-manufacturing facilities an onerous prospect, owing to its proclivity for slagging and inhibiting catalysts [99]. While tar has a detrimental influence on gas-manufacturing facilities, it is widely employed in other sectors and adds value. It is used to treat plant and human diseases and has a wide range of applications in the coatings industry [101]. The most prevalent use of liquid tar is in road construction [102]. In several states, especially developing ones, roads are constructed using a bitumen base and successive layers over it [103]. These layers get compressed over time, forming a robust and sturdy structure. These layers eventually erode and are replenished over time. The use of PW to coat bitumen is a technology that has seen a recent spike in popularity [104,105]. The benefits are twofold: non-biodegradable PW may be disposed of efficiently, and roads using PW have demonstrated greater performance than regular tar roads [106]. Nearly one ton of PW is used to pave one kilometer of road, resolving the complicated issue of garbage disposal and subsequent emissions [107]. It was discovered that using PW in the road construction process saves about $40,000 per kilometer of road [108]. India is the most vocal proponent of this technique, followed by the Netherlands and the United Kingdom. Other developing nations, such as Malaysia and Ghana, are also embracing the usage of PW in road construction [63].

The addition of PW results in a higher melting point for bitumen as well as greater flexibility. Roads with plastic in them have also been found to have a higher rainfall tolerance. Furthermore, the possibility of using a mixed plastic feed decreases the possible costs associated with PW segregation. Plastic roads have a higher level of ultraviolet resistance and have a longer lifespan. The stiffening effect of PW in bitumen is due to a gradual rise in the attraction forces among the bitumen and the PW over time. Furthermore, the application of oxidizing and linking agents improved the bonding forces even more [109]. In addition, the PW-modified road has been shown to carry heavier loads for lengthy periods of time [110].

### 4.6. Concrete Production

Concrete is widely used in the building sector and hence serves as a cornerstone of industrial advancement [111,112,113,114,115,116,117]. Recent concrete research has emphasized the use of various ingredients, with a focus on lighter materials, as alternatives to natural aggregates in concrete [118,119]. PW has the potential to be employed in the manufacture of concrete as an aggregate substitute [120,121,122,123]. Incorporating PW has a detrimental influence on concrete strength properties [124]. These recycled plastic aggregates, on the other hand, have the potential to enhance a variety of material characteristics and may be used in sound-insulating, thermal, and lightweight materials [25]. In structural concrete applications where lesser stresses are applied and durability is less crucial, a particular proportion of PW may be used [125]. Gu and Ozbakkaloglu [126] reported that PW is preferable for lightweight concrete manufacture. Additionally, because of its better functional performance, concrete comprising PW is suitable for thermal and soundproofing applications [127,128,129]. Due to the reduced conductivity of plastic and the higher porosity of composites containing PW as an aggregate, it was observed that composites manufactured with PW had a thermal conductivity five times less than normal composites [130]. Thermal conductivity reduced with increasing content due to the hydrophobicity of plastic, resulting in the creation of voids in the mixtures [131]. Numerous studies have revealed that PW, due to its porous nature, has the potential to be used as a sound-absorbing material in concrete [128].

**Figure 14:**
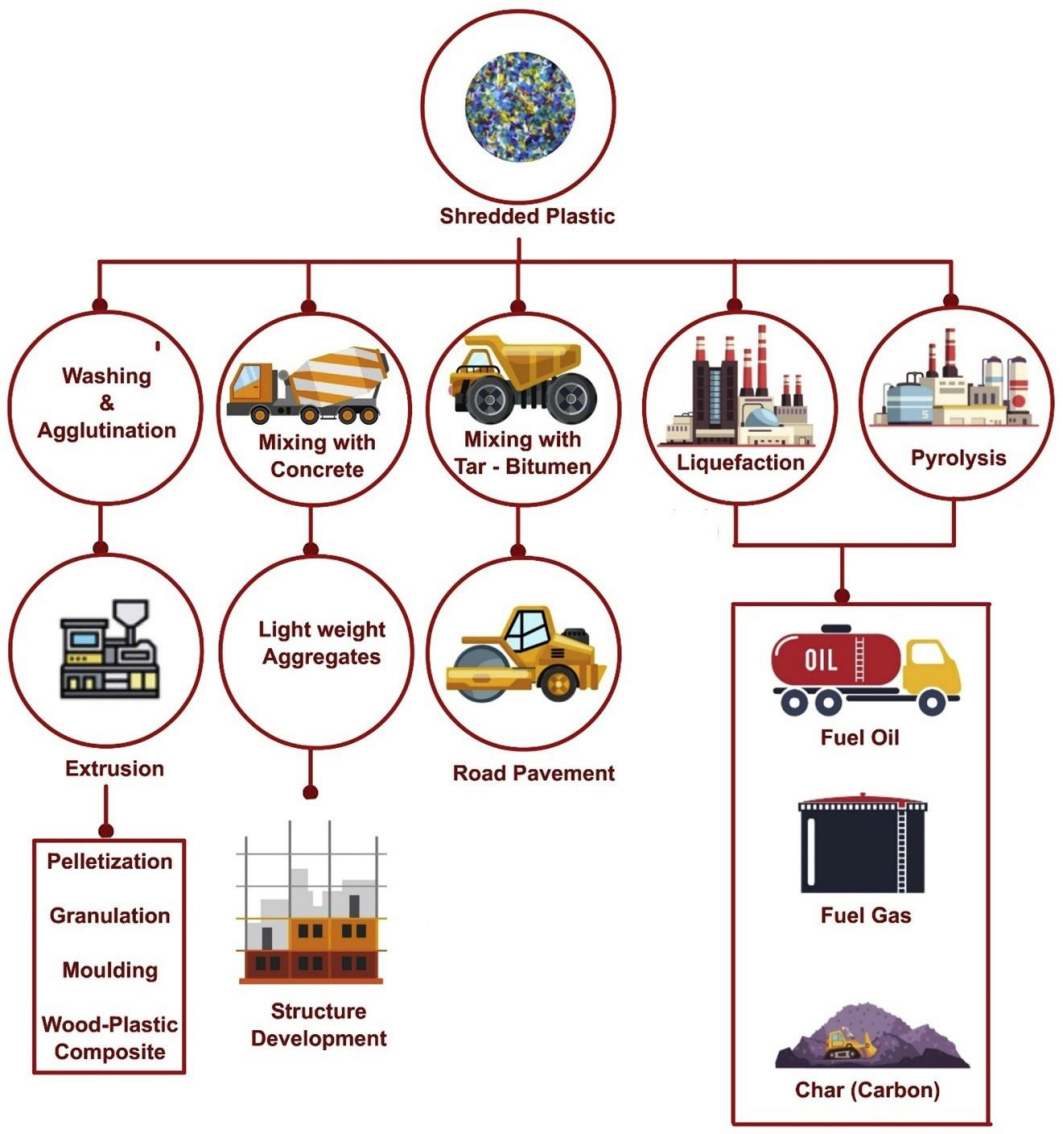
Flowchart of various management strategies for shredded plastic [63].

The use of PW in concrete will decrease not just dependency on natural resources but also manufacturing costs. However, using PW in large quantities in concrete is not recommended due to the considerable loss of strength. Usually, a replacement ratio of 10–15% of PW can result in a material with acceptable mechanical properties [132,133,134,135]. The increased air content and lower bonding capacity of plastic aggregates in concrete are the major causes of decreased strength. As a result, it is recommended that more research be conducted on the long-term behavior of plastic aggregates in concrete and their impact on the environment and service life. Various researchers have explored further usage of PW in construction, including plastic bottles in concrete blocks [136], plastic bottle bricks [137], and plastic fibers in concrete [138]. The management strategies for PW other than landfilling and their applications in various products are shown in Figure 14.

## 5. Discussion and Environmental Aspects

This study aimed to carry out a scientometric analysis of the different aspects of the literature on PW management up through 2021 and a review of the various management strategies for PW. The study identified six broad categories of PW management, i.e., landfilling, recycling, pyrolysis, liquefaction, road construction and tar manufacture, and concrete production. The impact of each management strategy on various aspects, such as land requirements, carbon emissions, energy requirements, costs, skilled labor requirements, localization, sustainability of the products, and impacts on society, was compared by constructing Table 6. After comparing all of the aspects, it was noted that landfilling is the least desirable method due to its negative impact on the environment and human health. On the other hand, the other methods benefit both waste management and the environment. Recycling, the other prominent current strategy of PW handling, was found to have nearly equal benefits and drawbacks. Pyrolysis and liquefaction are favorable since they produce important by-products like char and fuel and the prospect of energy recovery. When plastic-to-fuel methods are used, the reliance on fossil fuels for energy can be significantly decreased. However, the detrimental impact of the high energy needs and challenges with process localization raises concerns. The most effective methods of PW handling have been determined to be the conversion of PW into tar for road construction and into concrete for building construction. This is due to significant benefits, such as ease of localization, reduced greenhouse gas emissions, and the increased durability and sustainability of manufactured materials and constructed structures and roads, the existence of which considerably overcomes disadvantages like the inability to recover expended energy. Due to their extremely effective and sustainable advantages, these two strategies should be prioritized as alternatives to present strategies for future applications and research.

**Figure 15:**
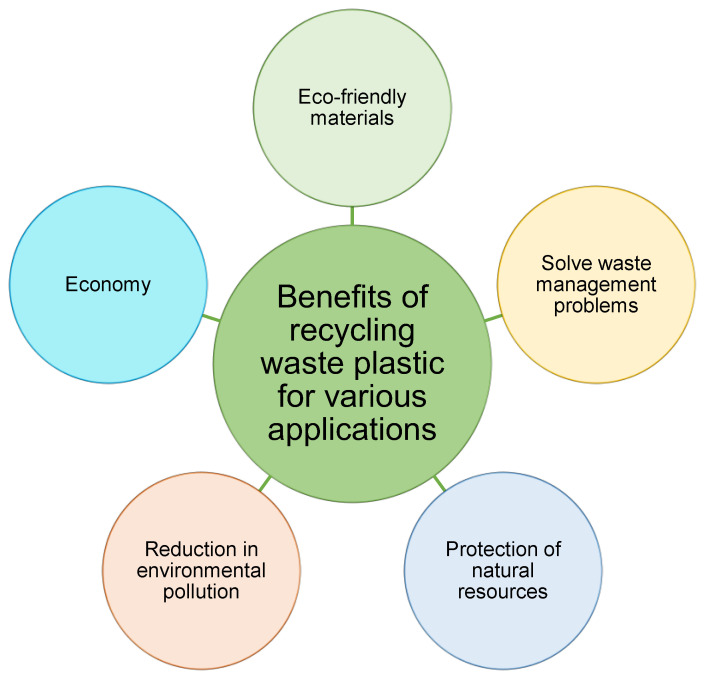
Advantages of plastic waste recycling and reuse.

As previously stated, a substantial amount of PW is created on a global scale. Currently, PW is dumped in landfills, burnt, or recycled, but present recycling processes are unsustainable, and PW dumping continues to be the most extensively utilized approach [139]. Additionally, the combustion of PW emits a significant quantity of CO_2_ into the environment [140]. On the other hand, recycling PW for various applications might alleviate the aforementioned difficulties related to its disposal in landfills. The benefits of reusing PW are illustrated in Figure 15. By decreasing the quantity of PW discarded in landfills, challenges for waste management may be mitigated while also safeguarding the natural environment. Due to the minimal or total lack of value of PW, its usage in the manufacture of various products decreases the expenditure of raw materials, thereby reducing the cost [141,142]. In addition, natural resources can be protected with the use of recycled PW. Thus, sustainable products might be manufactured at a cheaper cost by utilizing recycled PW.

## 6. Future Recommendations

This study discussed the various PW management strategies, their benefits and drawbacks, and environmental aspects associated with each strategy. After a comprehensive review, this study suggests the following:

- To control landfilling PW, local governments can promote closed-loop recycling of PW through a variety of initiatives and campaigns, as well as by imposing restrictions and fines on landfilling and incineration, while simultaneously lowering taxes on recyclable materials.
- Waste management techniques should be considered when designing plastics, mixes, and mechanical recycling processes. If the continuous use of plastics is required, it is better to understand their material life cycle and develop solutions that can sustain their worth over repeated uses and reprocessing. This uniformity will result in increased recycling rates, increased recycled content in products, and a reduction in the amount of plastic we export, landfill, and burn.
- Several aspects, including the state of the PW, the presence of impurities in the PW, and the type of reactor, influence the mechanism of catalytic pyrolysis and, therefore, the yield and distribution of the products. Thus, the effects of all these variables must be carefully known and regulated to assure the process’s viability. Another problem is developing standards for post-consumer PW processes and products, as well as adopting more complex pyrolysis technologies. Additionally, while it is possible to get a suitable product yield and composition at the laboratory scale, industrial producers will face difficulties maintaining the desired result while scaling up PW pyrolysis. If these obstacles are overcome, it will be possible to accomplish a low-cost, partial replacement of dwindling fossil fuels, as well as a reduction in PW, which is currently the primary source of environmental contamination, and a reduction in crude oil imports.
- Liquefaction of PW is a viable alternative to pyrolysis for resolving the problem of excess PW due to its gentler processing conditions [143]. Although the generation of oil from this process offers an alternative to the transportation industry’s reliance on fossil fuels, additional research is required to properly optimize the approach and determine the oil’s efficiency in engines.
- The use of PW in construction materials will reduce not only dependency on natural resources but also material costs. However, using PW as an aggregate in large quantities in construction materials is not recommended due to a significant loss of material strength. In general, a replacement ratio of 10–15% of PW may yield material with adequate mechanical properties [132,133,134,135]. The increased air content and lower bonding capacity of PW in concrete are the primary causes of decreased strength. Further research is required in this domain to optimize these aspects. By modifying the shape, size, and surface of PW particles, the material properties can be considerably improved. Additionally, guidelines for the use of PW in construction materials are crucial for reliable design and construction since they describe the appropriate content, allowable size and shape, and structural types. Indeed, standards are often formed over a period of several years after collecting sufficient, reliable information and an understanding of the subject has been achieved. As a result, it is advised that further research be conducted on the long-term performance of PW in construction materials, as well as tits impact on the environment and service life.

## 7. Conclusions

This study adopted a scientometric analysis strategy to assess various parameters of the available literature on plastic waste (PW) management. Bibliometric data of 6101 relevant articles were retrieved from the Scopus database, and analysis was carried out in VOSviewer software. Moreover, various management strategies for PW are discussed, along with their environmental aspects. The aim was to compare the benefits and drawbacks of all management strategies so as to recommend the most desirable ones. This study reached the following conclusions:

(1) The analysis of publication sources containing documents on the research of PW management revealed that the leading 3 sources are *Waste Management; Resources, Conservation and Recycling; and Science of the Total Environment* with 548, 270, and 227 papers, respectively. Moreover, the aforementioned journals also received the most citations in the related research area with 25,171, 13,142, and 11,555 citations, respectively.
(2) The evaluation of keywords in the subject research area disclosed that the top five most frequently occurring keyword combinations on the subject topic included “waste management”, “plastic, recycling”, “plastics”, and “plastic waste”. It was noticed that PW recycling has been under study mostly for waste management and sustainable development.
(3) The analysis of authors showed that only 60 authors had published at least 10 articles on PW management. The top authors, with respect to the number of articles, citations, and average citations, were categorized. In terms of total publications, the top 3 writers are Li, J. with 39; Wang, H. with 36; and Zhang, Y. with 34 publications. Thompson, R.C. leads the field in terms of citations with 7055; Wilcox, C. is second with 5130; and Li, J. is third with 1964 citations up through 2021. Additionally, when comparing average citations, the following writers stand out: Thompson, R.C. has about 588; Wilcox, C. has about 395; and Al-Salem, S.M. has about 140 average citations.
(4) The assessment of documents containing data on PW management showed that Jambeck, J.R. [55] obtained 4313 citations for their publication “Plastic waste inputs from land into the ocean”. Geyer, R. [13] and Hidalgo-Ruz, V. [56] received 3675 and 2007 citations, respectively, for their particular works and were rated in the leading 3. In addition, it was found that only 10 articles received more than 1000 citations on the subject through 2021.
(5) The leading countries, based on their participation in the research of PW management, were analyzed, and we discovered that only 48 countries produced at least 30 articles. The United States, India, and China each presented 871, 581, and 551 papers, respectively. However, the United States had the most citations (42,924), followed by the United Kingdom (30,071), and China (19,944).
(6) According to past studies, PW management strategies are broadly classified into six categories: landfills, recycling, pyrolysis, liquefaction, road construction and tar, and concrete production. Among these, landfilling is the most undesirable strategy as it causes environmental and human health concerns. On the other hand, recycling has equal merits and demerits; pyrolysis and liquefaction have more significant by-products, such as fuel and char, but they demand high energy. However, the use of PW for road construction and concrete production were found to be the most effective methods.
(7) Recycling PW to produce various products will result in sustainable solutions due to the prevention of the use of natural resources, the minimizing of waste management problems, the reduction of environmental pollution, and the production of eco-friendly materials at a lower cost.

## Figures and Tables

**Table 1:** Sources of publications with a minimum of 71 papers in the related field up through 2021.

S/N	Source	Documents	Total Citations
1	*Waste Management*	548	25,171
2	*Resources, Conservation and Recycling*	270	13,142
3	*Science of the Total Environment*	227	11,555
4	*Waste Management and Research*	221	4349
5	*Marine Pollution Bulletin*	213	7516
6	*Water Science and Technology*	136	3163
7	*Journal of Cleaner Production*	125	5024
8	*Environmental Pollution*	93	5108
9	*Journal of Hazardous Materials*	89	5470
10	*Chemosphere*	88	4762
11	*Environmental Science and Technology*	82	9656
12	*Bioresource Technology*	82	4222
13	*Environmental Science and Pollution Research*	78	1917
14	*IOP Conference Series: Earth and Environmental Science*	75	74
15	*Sustainability (Switzerland)*	74	698
16	*Journal of Environmental Management*	72	3106
17	*Water Research*	71	8495

**Table 2:** The leading 20 most utilized keyword combinations in the related study area.

S/N	Keyword	Occurrences
1	Waste management	3159
2	Plastic	2290
3	Recycling	1925
4	Plastics	1548
5	Plastic waste	1160
6	Waste disposal	1122
7	Solid waste management	709
8	Municipal solid waste	702
9	Wastewater management	656
10	Solid waste	636
11	Environmental impact	628
12	Polymer	618
13	Elastomers	572
14	Environmental monitoring	567
15	Plastic recycling	560
16	Waste treatment	548
17	Refuse disposal	509
18	Landfill	484
19	Wastewater treatment	480
20	Waste	465

**Table 3:** Authors with at least 10 publications in the related study field up through 2021.

S/N	Author	Documents	Total Citations	Average Citations
1	Li, J.	39	1964	50
2	Wang, H.	36	1153	32
3	Zhang, Y.	34	854	25
4	Wang, J.	30	873	29
5	Li, Y.	28	596	21
6	Wang, Y.	27	1417	52
7	Chen, X.	26	951	37
8	Wang, Z.	21	826	39
9	Liu, Y.	21	484	23
10	Li, X.	20	1085	54
11	Astrup, T.F.	20	610	31
12	Wang, S.	19	886	47
13	Wang, X.	19	479	25
14	Zhang, J.	18	553	31
15	Lee, J.	17	686	40
16	Wang, Q.	16	549	34
17	Walker, T.R.	15	937	62
18	Liu, X.	15	738	49
19	Rodgers, M.	15	297	20
20	Ragaert, K.	14	1124	80
21	Chen, Y.	14	695	50
22	Zhang, H.	14	688	49
23	Wang, C.	14	291	21
24	Zhang, L.	14	240	17
25	Wilcox, C.	13	5130	395
26	Al-Salem, S.M.	13	1817	140
27	Hardesty, B.D.	13	884	68
28	Christensen, T.H.	13	802	62
29	Zhao, J.	13	425	33
30	Kumar, S.	13	387	30
31	Kumar, A.	13	308	24
32	Thompson, R.C.	12	7055	588
33	Liu, H.	12	1038	87
34	Yang, J.	12	614	51
35	Wu, C.	12	610	51
36	Osibanjo, O.	12	569	47
37	De Meester, S.	12	456	38
38	Duan, H.	12	424	35
39	Arena, U.	12	416	35
40	Boldrin, A.	12	282	24
41	Mbohwa, C.	12	121	10
42	Mastellone, M.L.	11	438	40
43	Dewulf, J.	11	423	38
44	Zhang, C.	11	326	30
45	Liu, W.	11	188	17
46	Wang, L.	11	185	17
47	Rangel-Buitrago, N.	11	181	16
48	Li, M.	11	145	13
49	Hahladakis, J.N.	10	1236	124
50	Li, H.	10	599	60
51	Yang, Y.	10	484	48
52	Williams, P.T.	10	428	43
53	Xu, Z.	10	388	39
54	Zhang, X.	10	349	35
55	Fellner, J.	10	304	30
56	Kumar, V.	10	234	23
57	Rechberger, H.	10	208	21
58	Li, C.	10	130	13
59	Singh, S.	10	129	13
60	Kumar, R.	10	68	7

**Table 4:** Leading 10 most cited articles up through 2021 in the related study area.

S/N	Document	Title	Total Citations
1	Jambeck, J.R. [55]	“Plastic waste inputs from land into the ocean”	4313
2	Geyer, R. [13]	“Production, use, and fate of all plastics ever made”	3675
3	Hidalgo-Ruz, V. [13,56]	“Microplastics in the marine environment: A review of the methods used for identification and quantification”	2007
4	Teuten, E.L. [26]	“Transport and release of chemicals from plastics to the environment and to wildlife”	1449
5	Thompson, R.C. [57]	“Plastics, the environment and human health: Current consensus and future trends”	1268
6	Al-Salem, S.M. [58]	“Recycling and recovery routes of plastic solid waste (PSW): A review”	1191
7	Lebreton, L.C.M. [59]	“River plastic emissions to the world’s oceans”	1148
8	Hopewell, J. [60]	“Plastics recycling: Challenges and opportunities”	1123
9	Eerkes-Medrano, D. [61]	“Microplastics in freshwater systems: A review of the emerging threats, identification of knowledge gaps and prioritisation of research needs”	1082
10	Horton, A.A. [62]	“Microplastics in freshwater and terrestrial environments: Evaluating the current understanding to identify the knowledge gaps and future research priorities”	1031

**Table 5:** Leading 20 countries based on published documents in the present research area through 2021.

S/N	Country	Documents Published	Total Citations
1	United States	871	42,924
2	India	581	14,368
3	China	551	19,944
4	United Kingdom	443	30,071
5	Italy	348	10,416
6	Germany	289	12,161
7	Spain	269	9129
8	Japan	244	9415
9	Australia	231	13,314
10	Canada	208	8889
11	Brazil	187	5400
12	Malaysia	185	6305
13	France	174	5884
14	Indonesia	173	2547
15	Sweden	131	8164
16	South Korea	130	4353
17	Netherlands	128	8935
18	Poland	126	1809
19	Denmark	113	4722
20	Iran	110	2555

**Table 6:** The impact of various plastic waste management strategies on different aspects.

Management Strategy	Aspect							
	Land Requirement	Carbon Emissions	Energy Requirement	Cost	Skilled Labor Requirement	Localization	Sustainability of Product	Impact on Society
Landfills	A substantial area of useful land is converted into dumping sites	High carbon emissions due to incineration of plastic waste at landfill areas	Low energy requirement—only for equipment and transport	Cost-effective	No skilled labors required	Easily constructed and adopted anywhere	Difficult to keep landfills ecologically pleasant for an extended length of time	Pollutes the land and water; may result in the spread of infectious illness
Recycling	A small area of land is needed for a recycling plant	Moderate emissions during the conversion process	Moderate energy required for grinding/cutting	Expensive to convert one plastic item to another, and complete quantity is not converted	Skilled labor requirement is low, only required for segregation, cleaning, and sanitation	Easily adopted anywhere on preferred scale	Favorable influence, as plastic items are continuously transformed into other plastic products, but the chemical structure of the plastic remains constant	Prevents hazardous plastic waste from being disposed of by transforming it into other useful materials.
Pyrolysis	A small area of land is needed for a pyrolysis reactor	Low levels of carbon emissions since the process is oxygen-independent	High energy required to maintain high temperature and pressure	Highly expensive equipment and reactor as well as process	Very skilled labor required for design and supervision of reactor	Not adopted easily due to high complexity and cost	No significant impact since pyrolysis products are unlikely to remain in their original state for an extended period	Produces highly valuable products such as liquid and gaseous fuels, as well as char, which enables the problem of overdependence on current fossil fuel stocks to be resolved.
Liquefaction	A small area of land is needed for a hydrothermal reactor	Low levels of carbon emissions due to the absence of oxygen in the products	High energy required for efficient thermal degradation	Highly expensive equipment and water consumption	Very skilled labor required for design and supervision of reactor	Not adopted easily due to high complexity and cost	No significant impact since liquefaction products are unlikely to remain in their original state for an extended period	Produces liquid fuels and charcoal, both of which are extremely important and are employed in the generation of energy.
Road construction and tar	A small area of land is needed for a mixing plant	Low levels of carbon emissions during plastic and tar mixing	Low energy required for mixing	Low cost for mixing plastic and tar	Very low requirement	Might be adopted on a small scale for private roads	Favorable impact since roads built with plastic waste used in the manufacturing of tar remain for an extended length of time without deterioration	Increases the availability of raw materials for tar production and gives economic benefits
Concrete production	A small area of land is needed for a mixing plant	Very low levels of carbon emissions during plastic mixing in concrete	Very low energy required for mixing plastic in concrete	Very low cost required for mixing plastic in concrete	Very low requirement	Might be adopted on a small scale for private buildings	Favorable impact since buildings constructed using plastic in the concrete mix have a better service life.	Provides raw materials for building construction, hence preventing the development of home and municipal plastic waste.

## Data Availability

Not applicable.
